# Exploring Quantum Computing for Metal Cluster Analysis

**DOI:** 10.1021/acs.jpca.5c01404

**Published:** 2025-06-27

**Authors:** Nia Pollard, A’Laura C. Hines, Andre Z. Clayborne

**Affiliations:** † Department of Chemistry and Biochemistry, 3298 George Mason University, Fairfax, Virginia 22030, United States; ‡ Quantum Science and Engineering Center, 3298 George Mason University, Fairfax, Virginia 22030, United States

## Abstract

This study explores the application of quantum computing to metal cluster analysis through the development and implementation of a quantum-DFT embedding workflow. Classical computational methods, while transformative, often face limitations in achieving chemical accuracy and computational efficiency, particularly for nanoscale systems. To address these challenges, we integrate the Variational Quantum Eigensolver (VQE) with density functional theory (DFT), leveraging the capabilities of quantum computing aiming to improve the modeling of electronic structures. Aluminum and gold clusters were used as model systems to test the established workflow. The workflow successfully determined electronic properties for aluminum clusters up to Al_7_^–^. Although gold clusters were used as a test case to investigate the potential reduction of nitric oxide (NO), memory limitations, the lack of relativistic corrections, and the inability to handle open-shell systems presented challenges that underscore the need for advancements in quantum hardware and algorithms. This proof-of-concept study demonstrates the potential of quantum DFT embedding to advance materials discovery, including applications in catalysis and nanomaterial design, while providing insights into the current limitations of near-term quantum devices.

## Graphical abstract

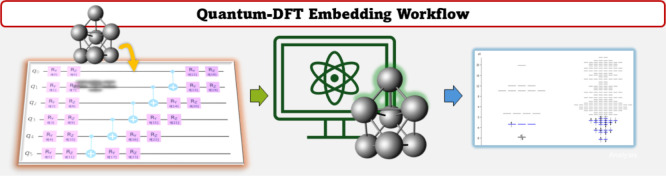

## Introduction

Since the advent of classical computers, computation has played a pivotal role in advancing chemistry. In nanochemistry specifically, classical computing methods such as density functional theory (DFT) and time-dependent density functional theory (TDDFT) have revolutionized the way researchers analyze various properties of nanoscale systems. Despite these advancements, classical computing methods sometimes fall short of achieving both chemical accuracy and computational efficiency when simulating nanoscale systems.^1–5^ As the number of atoms in a system increases, the computational cost grows disproportionately, while the efficacy of classical methods diminishes. Nanoparticle systems, which often comprise hundreds to thousands of atoms, pose significant challenges for electronic structure investigations when using classical approaches. To address this, researchers frequently “reduce” or simplify nanoparticle systems with ligands to gain insights into their electronic structure.^6–11^ While these simplified models often align with experimental results, they can introduce shifts of 0.2–0.5 eV in UV–vis spectra.^6,11^ Thus, there is an urgent need for methods that deliver improved chemical accuracy with greater computational efficiency across various size scales.

While classical computing has been transformational in nanochemistry, its limitations in computational efficiency and accuracy become pronounced at larger scales, particularly in highly correlated systems. Quantum computing, which leverages quantum phenomena such as superposition and entanglement, offers a promising solution to these challenges by inherently simulating quantum mechanical systems. As quantum systems themselves, quantum computers are uniquely suited for simulating quantum mechanical problems without the exponential memory limitations faced by classical systems.^12^ To date, research efforts have focused on quantum algorithm development and building quantum computing architectures.^13–16^ However, applications of quantum computing to chemical problems at the nanoscale remain limited. For example, Rossmannek and colleagues used quantum computing to investigate the electronic structure of small molecules.^17^ Similarly, Choudhary applied quantum computing to simulate electron and phonon band structures using quantum algorithms for materials applications.^18^ While quantum computing holds the promise of significantly accelerating calculations traditionally performed classically, current quantum devices–often referred to as NISQ (noisy intermediate-scale quantum) devices face several limitations.^15^ Existing quantum algorithms can also be inefficient when simulating chemical systems due to the substantial resources required to demonstrate quantum advantage.^19^ For example, simulating a simple chromium dimer would require at least one million physical qubits—far exceeding the capabilities of current quantum computers.^19^

Among the earliest quantum algorithms applied to chemical systems was quantum phase estimation (QPE), introduced by Alexei Kitaev to study the Abelian stabilizer problem.^19–21^ QPE was later extended to estimate the phase of an arbitrary unitary operator, a form commonly utilized today. By leveraging phase kickback and the inverse quantum Fourier transform (IQFT), QPE extracts the binary representation of a phase or eigenvalue.^22^ This process is crucial for various quantum algorithms, including Shor’s algorithm for factoring large numbers and algorithms for solving linear systems of equations.^22^ The essence of QPE lies in its ability to estimate phases with high precision, utilizing quantum superposition and interference to achieve exponential speedup compared to classical methods. Researchers anticipate that QPE will demonstrate quantum advantage once large-scale fault-tolerant quantum computers are developed.^23^ However, QPE requires millions of qubits even for relatively small systems, far surpassing the capabilities of current NISQ devices.^19^ An alternative approach, the variational quantum eigensolver (VQE), has been proposed to address the limitations of QPE, offering compatibility with near-term quantum hardware.^24,25^ VQE combines quantum and classical resources to to find variational solutions to eigenvalue problems. The method typically begins with a trial wave function derived from a classical computation, such as Hartree–Fock or DFT. This wave function is parametrized using a quantum circuit, known as an ansatz, whose parameters are iteratively optimized by a classical algorithm to minimize the energy expectation value. This process approximates the ground state energy of the system while leveraging the variational principle, which guarantees the measured energy is always an upper bound to the true ground state energy. The hybrid nature of VQE, where quantum computers evaluate Hamiltonian expectation values and classical computers adjust parameters, mitigates quantum noise and errors—challenges inherent to current NISQ devices. This synergy is particularly advantageous in the NISQ era, where limited coherence times and error correction remain significant barriers. VQE’s flexibility to run on any quantum device, coupled with its potential for error suppression, makes it a compelling candidate for benchmarking early quantum computers.^25^ According to McLean, VQE’s intrinsic robustness to quantum errors, combined with its low coherence time requirements, positions it as a potential algorithm to surpass classical computing for specific problems.^25^ While VQE simulations for small molecules have been performed on various quantum computing platforms,^26–30^ little to no work exists applying this method to the nanoscale. This work, in which we develop a quantum-DFT embedding workflow for the investigation of metal subnanometer clusters, offers a perspective on the current capabilities of present-day quantum computers for simulating the electronic response properties of these systems.

Beyond their suitability for current quantum computing capabilities, metal clusters such as aluminum and gold are of significant interest due to their tunable electronic, optical, and catalytic properties.^3,7,10,31–33^ For example, gold clusters exhibit unique reactivity at the nanoscale and have been extensively studied for applications in catalysis,^34,35^ medicine,^36,37^ and nanoelectronics^38,39^ while aluminum clusters serve as lightweight alternatives with potential relevance in materials design and energy storage.^40,41^ Understanding their electronic structure is key to tailoring their function in these emerging technologies. Therefore, developing scalable and accurate quantum-classical workflows to model such clusters not only advances algorithmic capabilities but also supports broader efforts in nanomaterials discovery and design.

## Methods

Building on the foundation of VQE, we developed a quantum-DFT embedding workflow to overcome the challenges faced by classical methods in modeling highly correlated systems at the nanoscale. This workflow integrates quantum mechanics, as implemented through VQE, into the established DFT framework, enabling a more accurate investigation of the electronic structure of metal clusters. Using this approach, we implemented our quantum-DFT embedding workflow (Figure 1) on Qiskit (Version 43.1), IBM’s open-source platform for quantum computing.^42^ Quantum-DFT embedding is a computational strategy that combines the strengths of quantum mechanics and DFT to study complex systems with greater accuracy and efficiency. This method partitions a system into distinct regions, applying varying levels of computational rigor based on each region’s significance to the overall properties of the system. By incorporating VQE into the DFT embedding framework, this approach leverages the unique capabilities of quantum computing to address classical DFT’s limitations in capturing strong electron correlations. This hybrid methodology holds significant potential for investigating complex materials and molecular systems that are otherwise difficult to model accurately using classical methods alone. By integrating quantum computing into nanoscale studies, the quantum-DFT embedding workflow represents a step forward in the application of quantum algorithms to chemistry.

**Figure 1:**
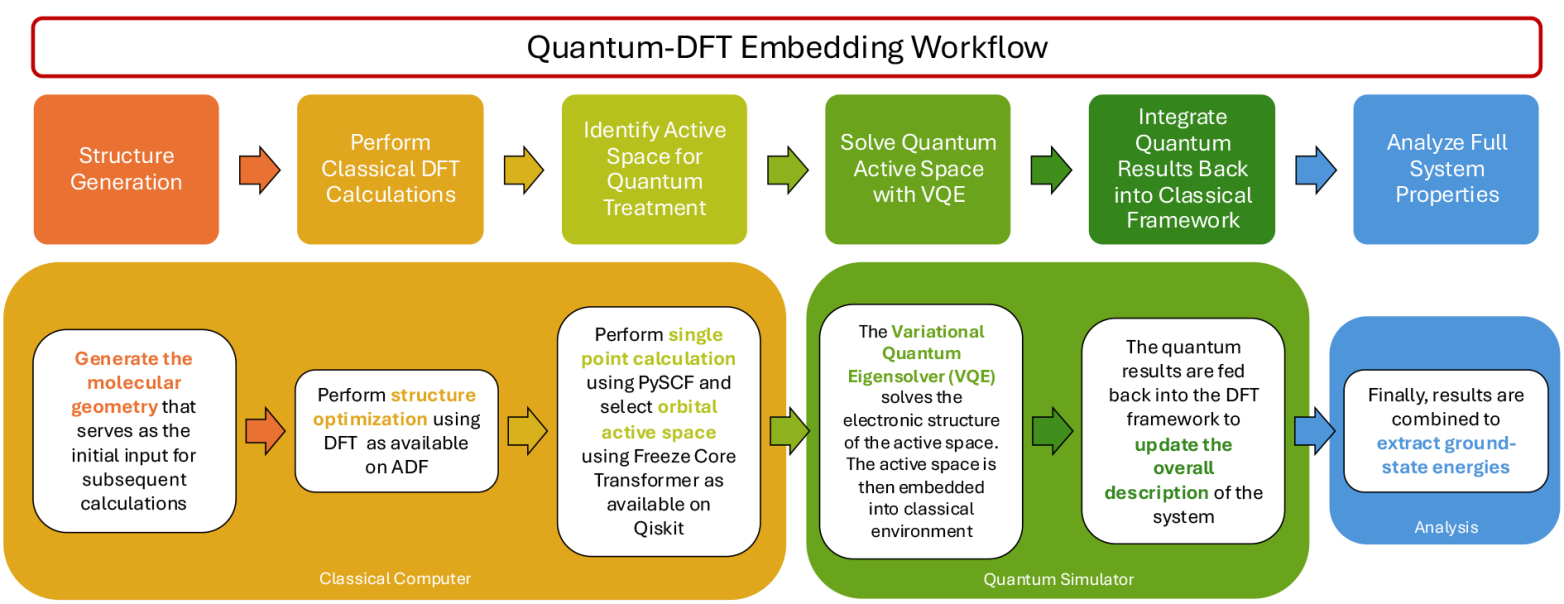
Developed quantum DFT-embedding workflow.

A preliminary step in the developed workflow is identifying viable materials for study at the nanoscale, which can range from 2D to 3D materials. Structures may be sourced through literature review, collaborations, or generated using any molecular visualization software. In this work, we apply our workflow to aluminum structures that were designed utilizing the Amsterdam Density Functional (ADF)^43^ program, to compute their ground-state energies. After generating the structures, DFT calculations are performed to determine orbital occupancy, which is used to identify the active space—or “active” orbitals—of the system. Active orbitals are those most ″energetically important″ to the electronic structure calculation. For example, in catalytic systems, active orbitals are those undergoing the most significant changes during interactions with the studied molecule. Once the active space is defined, the Fermionic Hamiltonian is mapped to qubits using a mapping technique such as the Jordan-Wigner Mapper. Subsequently, an ansatz is generated. An ansatz is a parametrized quantum state designed as an approximate solution to the quantum problem. It serves as a trial wave function, constructed with a defined structure and adjustable parameters, which are optimized to minimize the energy expectation value of the Hamiltonian. The choice of ansatz is critical, as it determines the algorithm’s expressiveness and efficiency.^44^ The expectation value of the Hamiltonian is then measured on the quantum computer, and the ansatz parameters are variationally adjusted until the energy is minimized. This iterative process continues until convergence is achieved, yielding the electronic structure data for the system. While this workflow aligns with the typical scheme of the VQE algorithm, to our knowledge, neither quantum-DFT embedding nor VQE has been previously applied to chemical systems at the nanoscale. This work serves as a proof-of-concept study, demonstrating the potential utility of quantum computing for investigating metal clusters.

Bare, closed-shell aluminum clusters are ideal candidates for the developed quantum-DFT embedding workflow. These clusters have been extensively studied using both DFT and experimental methods, offering a range of shapes and sizes while prominently featuring stable closed-shell systems. In its current state, the developed workflow only allows for an active space and inactive space with an even number of electrons, limiting its applicability to systems with unpaired electrons. However, further developments to Qiskit beyond version 43.1 may enable the inclusion of such systems within this embedding framework.

All aluminum clusters, Al_3_^–^ to Al_8_ (Figure 2), were designed and preoptimized using the ADF program^43^ (please see Supporting Information for preoptimization details). To accommodate the active space restriction, systems with an odd number of electrons were assigned an additional negative charge. Geometry optimizations were performed at the density functional theory (DFT) level using the gradient-corrected Perdew, Burke, and Ernzerhof (PBE) exchange-correlation functional. This functional, frequently utilized by our group and others, provides reliable results for small to medium-sized aluminum clusters. During optimization, all atomic positions were allowed to relax freely until the forces were less than 0.05 eV/Å, ensuring the structures achieved their lowest energy configurations. Single-point energy calculations were performed on the preoptimized aluminum clusters using the open-source code PySCF.^45–47^ We employed the default functional, local density approximation (LDA)^48^ and a minimal 6-31g basis set. We note that LDA is the default functional available within the PySCF driver on Qiskit Version 43.1.^42^ After the initial classical calculations determined orbital occupancy, an active space of four electrons and three orbitals (two occupied and one unoccupied close to the Fermi level) was selected. The reduced Hamiltonian was then computed and mapped to qubits via the Jordan–Wigner mapping technique. This method encodes the quantum states of Fermionic systems, such as electrons in atoms and molecules, into qubits for quantum simulation. All simulations were carried out using the Statevector Simulator, which allowed us to calculate the VQE energies and assess the capabilities of our workflow under ideal conditions. Also, an EfficientSU2 circuit was employed as the ansatz. The Statevector Simulator provided a valuable baseline for evaluating the workflow, demonstrating its performance in the absence of noise.

**Figure 2:**
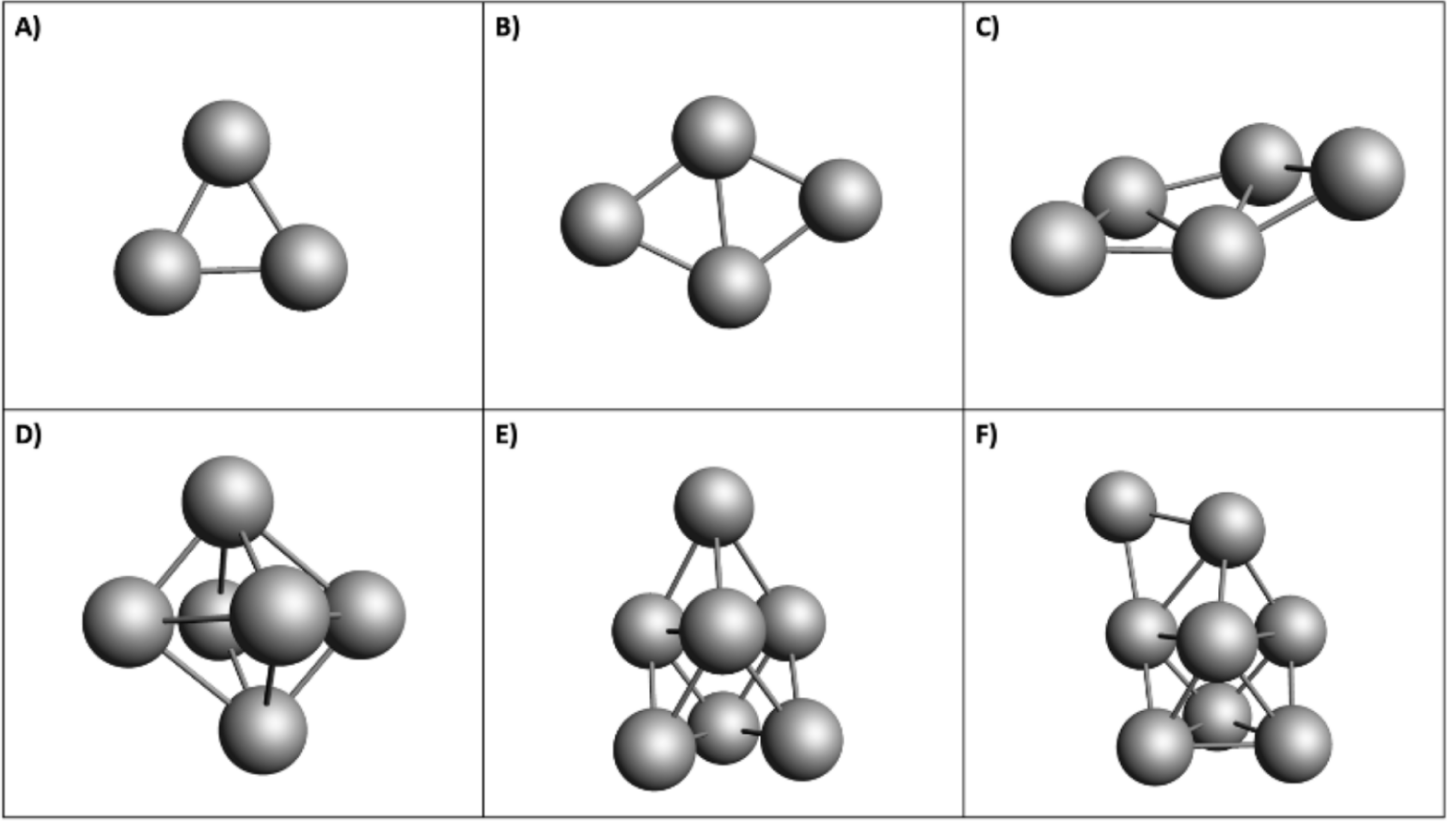
Optimized geometries of bare aluminum clusters, Al_3_^–^ to Al_8_. Al: aluminium.

In addition to aluminum clusters, we applied the same quantum-DFT embedding workflow to gold clusters, which posed greater computational challenges that are later outlined in the following sections. Following the steps of the developed workflow, structures were generated and optimized following a similar procedure as applied to the aluminum systems (Section IV). We began by testing the workflow with two small, bare gold cluster systems, Au_4_ and Au_13_(Figure 3). Au_4_ was generated using the program Avogadro.^49^ Au_13_ was obtained from literature; it is the core structure of a commonly studied gold nanoparticle system, Au_25_.^50^ All calculations on the bare gold clusters were carried out using the open-source code Quantum Espresso.^51–53^ Geometry optimizations were again performed at the DFT level of theory using the gradient-corrected PBE^54^ exchange-correlation functional. All atoms were allowed to freely relax until the forces were smaller than 0.05 eV/Å, enabling structures to achieve their lowest energy configurations. We employed ultrasoft pseudopotentials and a plane-wave basis set. The cutoff energy for the expansion of the plane-wave basis was set to 75.0 Ry. We used a k-point set of 2 × 2 × 1 for the integration of the Brillouin zone. The occupation of the Kohn–Sham eigenstates was smeared by Gaussian smearing to aid SCF convergence.

**Figure 3:**
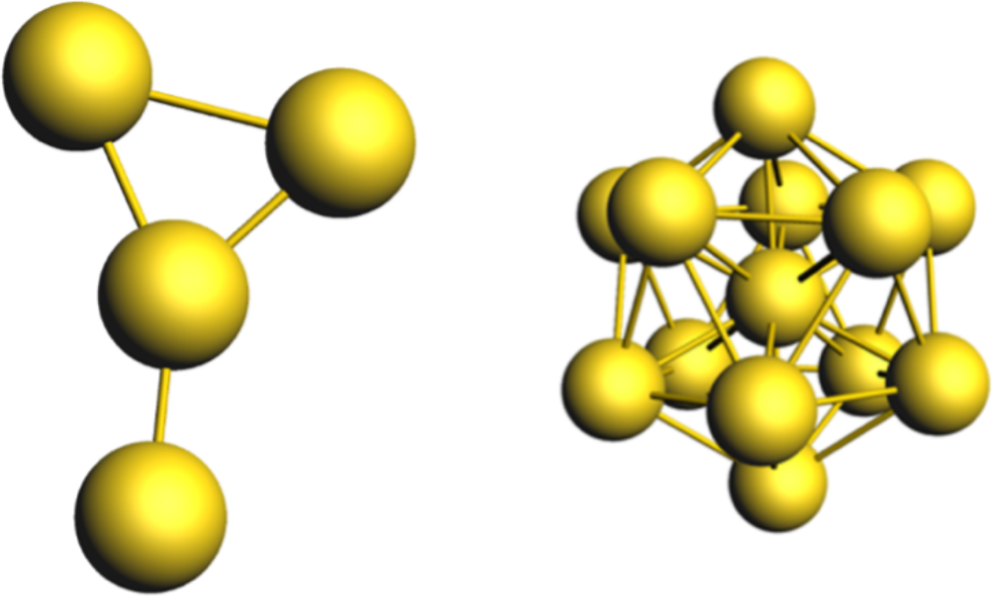
Optimized geometries of bare gold clusters Au_4_ and Au_13_. Au: gold.

## Results and Discussion

### Aluminum Clusters

When employing our quantum-DFT embedding workflow on the bare aluminum clusters, we reached the memory limit on the Qiskit cloud (8192.00 MB) with the cluster composed of 8 aluminum atoms. Therefore, we could only obtain the ground state energy of the aluminum clusters with up to 7 atoms (Table 1). The ground state energies of the aluminum clusters show a clear trend of increasing magnitude as the system size grows, aligning with theoretical expectations. This demonstrates the capability of the quantum-DFT embedding workflow to capture size-dependent properties of nanomaterials. Additionally, the convergence of ground-state energies with increasing active space size highlights the critical role of active space selection in balancing computational cost and accuracy (Table 2). These findings highlight the workflow’s potential for modeling nanoscale systems and emphasize the scalability challenges posed by current quantum computing platforms, particularly regarding memory limitations. Future advancements in quantum devices, such as increased memory capacity and improved qubit coherence, could enable the application of this methodology to larger systems. This work not only validates the use of VQE for nanoscale systems but also sets the stage for practical applications in materials design. Potential applications include developing aluminum-based catalysts and exploring superatomic systems, demonstrating the promising intersection of quantum computing, materials science, and chemistry.

**Table 1:** Ground State Energies Obtained from Qiskit Statevector Simulator (Al_3_^–^ to Al_7_^–^)

**System**	**Total Ground State Energy (Hartree)**
Al_3_^–^ (**A**)	–725.60
Al_4_ (**B**)	–967.42
Al_5_^–^ (**C**)	–1209.37
Al_6_ (**D**)	–1451.17
Al_7_^–^ (**E**)	–1693.13

**Table 2:** Ground State Energy of Al_4_ with Varied Active Space

**Electrons Included**	**Total Ground State Energy (Hartree)**
4 electrons, 3 orbitals	–967.420086
6 electrons, 4 orbitals	–967.420169
8 electrons, 5 orbitals	–967.429366
10 electrons, 6 orbitals	–967.429367

While the workflow successfully simulated up to 7 aluminum atoms, we investigate the role of active space size by increasing the number of orbitals included for Al_4_ (Table 2). We selected this system for the active space variation study because it is a chemically reasonable system that avoids complicating factors such as orbital hybridization.^55^ Its relatively simple electronic structure allows for clearer evaluation of the workflow’s behavior with respect to active space size, while remaining computationally feasible for near-term quantum simulations. The workflow was successful with obtaining the ground state energy of Al_4_ with an active space of up to 10 electrons (6 orbitals), but Qiskit cloud failed when attempting to simulate an active space of 12 electrons (7 orbitals), likely due to memory limitations. With increased memory, larger active spaces could potentially be explored. This highlights how the size of the active space also plays a role on computational resources needed. Lastly, as we included more electrons in the active space, the total ground state energy converged to a final value of −967.43 hartree. We note that this study is focused on demonstrating the feasibility of the workflow. Accuracy benchmarking against published or high-level reference data will be addressed in a follow-up study.

### Gold Clusters

To determine the lowest-energy structure of Au_4_, we analyzed several initial configurations (Figure 4) and computed their energy values. Our results identified the ″Y-shaped″ structure as the lowest-energy configuration, consistent with previous studies.^56^ After identifying the lowest-energy structure, we investigated its interaction with an NO molecule. The NO molecule was preoptimized using the same methodology. Interaction points on the cluster were chosen randomly, and the entire system was fully relaxed during geometry optimization. Despite testing multiple interaction sites, we found that Au_4_ did not weaken the N–O bond (Figure 5). During optimization, the NO molecule readjusted to its original double-bond length and moved further away from the cluster. These results suggest that Au_4_ alone may require additional hydrogen atoms to facilitate catalytic activity. Also, while our focus was on breaking the N–O bond, to investigate and identify a true catalyst, a comprehensive investigation of thermodynamic properties is necessary.

**Figure 4:**
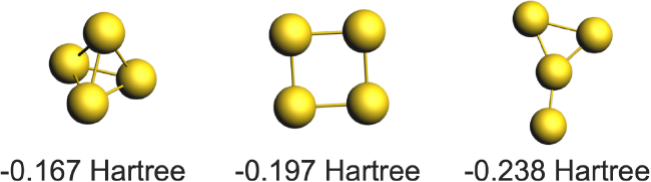
Optimized geometries and ground-state energies of different 4-atom gold clusters. ″Y-shaped″ structure is the lowest-energy configuration. Au: gold.

**Figure 5:**
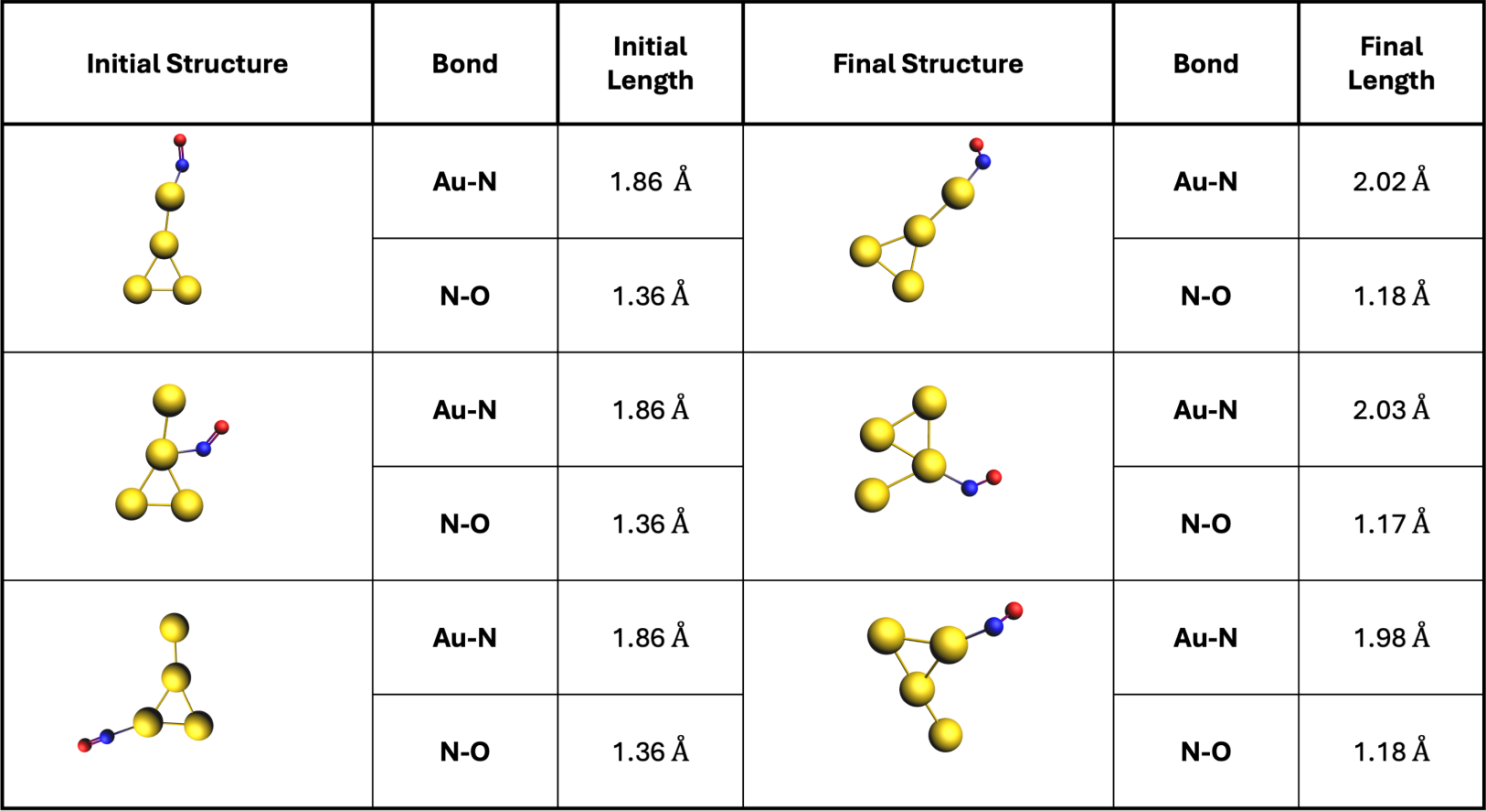
Initial and final configurations of Au_4_+NO. At all interaction points, Au_4_ did not weaken the N–O bond. The NO molecule readjusted to a double-bond bond length (1.14 to 1.17 Å) and moved further away from the cluster. Au: gold, N: blue, O: red.

Although the planar structure of Au_13_ is known to be more stable than the icosahedral structure^31^ we chose to study the icosahedral Au_13_ due to the significant role symmetry plays in electronic response properties. Systems with higher symmetry are often associated with enhanced catalytic activity. After optimizing the icosahedral Au_13_ structure, we tested its interaction with an NO molecule. Given the cluster’s symmetry, all points on the surface are equivalent, so only one active site was examined. Upon bonding with the cluster, the NO molecule underwent geometry optimization, and the entire system was relaxed. The results showed that the N–O bond stretched significantly, with the oxygen–nitrogen distance exceeding 3.0 Å. These findings suggest that Au_13_ could serve as a potential catalyst for NO reduction. However, it is important to note that the icosahedral structure is not the lowest-energy configuration for Au_13_. Furthermore, the precise thermodynamics of potential reaction pathways to form N_2_ or NH_2_ have not yet been explored, requiring further investigation to confirm catalytic viability.

Due to its ability to stretch the N–O bond (Figure 6), Au_13_ was the ideal candidate for testing our workflow. After the Au_13_+NO system was fully relaxed, the next step involves utilizing DFT to gain insight into the orbital occupancy and determine the active space. We hypothesized that the uppermost orbitals of Au_13_ would be considered the most “active”. We confirmed our hypothesis by visualizing the electron density of Au_13_+NO. We found that the gold atom connected to the nitrogen appeared to be sharing the most electron density with the NO molecule (Figure 7). We also obtained quantitative data on how the orbitals were filled before and after the addition of the NO molecule (Tables 3 and 4). We found that only the gold atom attached to the nitrogen underwent a large change in electron density (over 5%) when the NO molecule was attached, while the surrounding gold atoms changed by less than 2%. This further confirmed our hypothesis on our active space.

**Figure 6:**
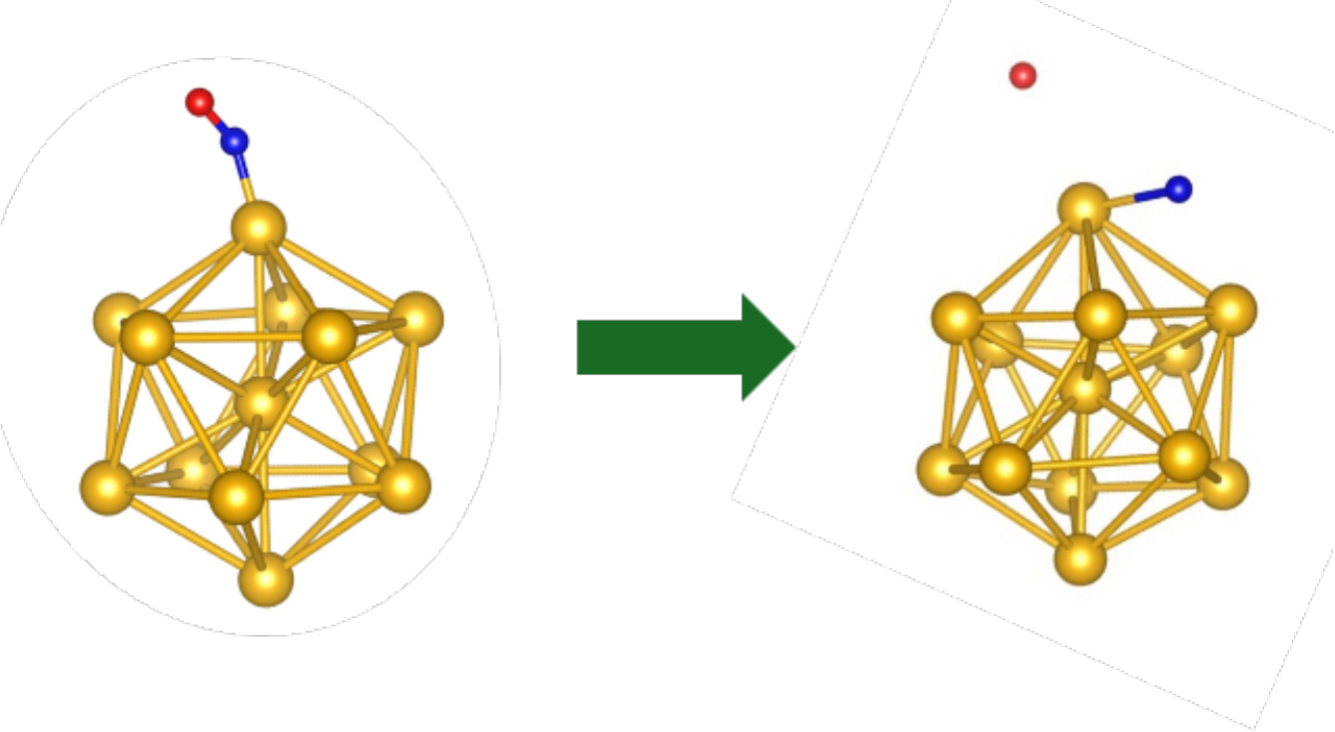
Au_13_ extends the N–O bond. Au: gold, N: blue, O: red.

**Figure 7:**
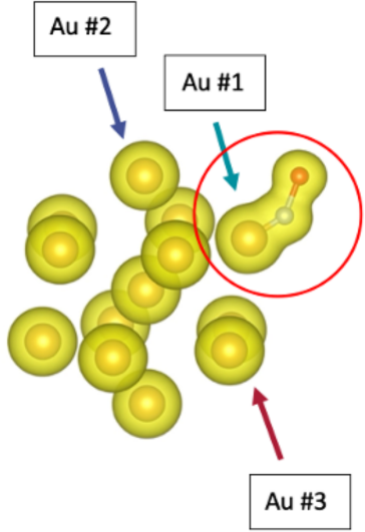
Visualization of the electron density of Au_13_+NO.

**Table 3:** Lowdin Charge Analysis of Au_13_ before the Addition of NO

	**Total charge**	**s**	**p**	**d**
Au #1	10.7418	1.4298	1.4898	7.8223
Au #2	10.7443	1.4298	1.4921	7.8224
Au #3	10.7401	1.4300	1.4879	7.8223

**Table 4:** Lowdin Charge Analysis of Au_13_+NO

	**Total charge**	**s**	**p**	**d**	Δ%
Au #1	10.1631	1.0251	1.5531	7.5849	**5.39%**
Au #2	10.8674	1.4375	1.5331	7.8969	1.15%
Au #3	10.7829	1.4400	1.4451	7.8978	0.40%

Once information on how the orbitals were filled was obtained, the next step of the workflow is to model the active space of this system utilizing the VQE on Qiskit.

#### Challenges of Modeling Gold Clusters on Qiskit

Modeling the Au_13_+NO system using the developed workflow presented several challenges. One significant issue was the presence of partially occupied orbitals, which are common in systems exhibiting strong correlation, especially in catalytic systems involving transition metals like gold. These orbitals could not be manually specified within Qiskit’s PySCF driver, which restricts flexibility in defining custom active spaces. To accurately capture the physics of such systems, it may be necessary to use a third-party program, such as ORCA^57^ or ADF, to extract wave functions that could serve as initial points for quantum computing calculations. These programs offer more advanced capabilities for orbital analysis, including tools to export natural orbitals and occupation numbers. These orbitals can be analyzed to manually define an active space, and molecular integrals can then be converted into a second-quantized Hamiltonian using tools like OpenFermion^58^ before mapping to a qubit Hamiltonian for use in Qiskit. However, we chose to utilize Qiskit’s built-in PySCF driver for this study due to its efficiency, ease of integration with Qiskit, and its ability to automatically generate the Hamiltonian, making it the most practical option for near-term applications. While the PySCF driver greatly streamlined parts of our workflow, modeling gold systems presented additional challenges. The large number of electrons in gold and memory constraints on Qiskit’s cloud infrastructure limited our ability to study these heavy atoms effectively. Additionally, the PySCF driver did not incorporate relativistic effects, which are essential for accurately modeling gold systems. Another limitation of our methodology is the requirement that both active and inactive space electrons be an even number, making open-shell systems with unpaired electrons difficult to model using this approach.

Frequent updates to Qiskit’s libraries also posed challenges. As a powerful and continuously evolving open-source platform, Qiskit’s regular updates reflect its commitment to innovation and providing cutting-edge tools for quantum computing. However, these updates occasionally resulted in deprecated functionalities, which can impact existing workflows. While researchers can use earlier versions of Qiskit to maintain compatibility with deprecated code, it is recommended to use the latest version to ensure optimal performance on quantum hardware. These developments highlight the importance of maintaining accessible and up-to-date tutorials to support researchers in adapting to these changes and applying quantum computing to chemical systems effectively.

Despite the current limitations of our workflow, this study provides a critical proof-of-concept that showcases the potential of quantum computing for tackling the complexities of nanochemistry.

## Conclusions

This study demonstrates the feasibility and potential of employing quantum-DFT embedding workflows, specifically utilizing VQE, to investigate the electronic structure and catalytic properties of metal clusters. Our investigation highlights the promises and current limitations of quantum computing in the broad field of nanochemistry. We developed a comprehensive quantum-DFT embedding workflow that integrates classical DFT and quantum variational approaches on Qiskit, IBM’s open-source quantum computing platform, to analyze metal clusters. The workflow was tested on gold and aluminum nanoclusters, highlighting its versatility and adaptability to different materials. Despite efforts to analyze gold clusters, significant challenges arose. These included the inability of Qiskit to handle partially filled orbitals manually, memory limitations for heavy atoms like gold, and the absence of relativistic effects in PySCF. Additionally, the frequent deprecation of Qiskit libraries posed hurdles in maintaining the workflow’s functionality. These challenges emphasize the need for advancements in quantum computing tools and algorithms to effectively handle heavy and complex systems. However, by focusing on aluminum clusters, a system with “lighter” atoms that require no relativistic corrections, we were able to successfully apply our workflow. We demonstrated the ability to compute the ground-state energies for aluminum clusters up to Al_7_^–^, utilizing an active space approach to balance computational resources and accuracy. Lastly, we illustrated that increasing the active space for aluminum clusters improved the accuracy of ground-state energy calculations but also demanded higher computational resources. This emphasizes the critical role of active space selection in quantum chemical calculations, especially given the memory constraints of current quantum computing platforms.

To fully realize the potential of quantum computing in nanoscience, several advancements are necessary including enhanced quantum hardware, algorithm development, etc. Collaborative efforts from chemists, physicists, and computer scientists will also be key in overcoming current limitations and driving forward the capabilities of quantum simulations in chemistry. While the field of quantum computing for nanochemistry is still in its infancy, this work demonstrates quantum computing’s potential to revolutionize the study of metal nanoclusters. With continued advancements and interdisciplinary collaboration, quantum computing can provide new insights into the electronic structure and catalytic properties of complex nanoscale systems. We hope this serves as a foundation for others to pursue using quantum computing for clusters and other nanoscale systems.

## Supplementary Material



## Notes

ADF computational details; HOMO and LUMO of Al_4_ (Figure S1) (PDF)
