# Supplementary material for: Exploring Quantum Computing for Metal Cluster Analysis
Source: J Phys Chem A. 2025 Jun 27;129(27):5923–30. doi: 10.1021/acs.jpca.5c01404 (PMC12257506; doi:10.1021/acs.jpca.5c01404)
Supplement: Supplementary file 1 [file jp5c01404_si_001.pdf]

Exploring Quantum Computing for Metal Cluster Analysis

Nia Pollard, A'Laura C. Hines, and Andre Z. Clayborne*

Department of Chemistry and Biochemistry, George Mason University, 4400 University Dr, Fairfax, VA 22030, United States of America

E-mail: aclaybo@gmu.edu

Supporting Information

ADF Computational Details

Initial aluminum cluster geometries were designed based on a previous study of aluminum ground state geometries.[1] Calculations were carried out using the DFT Amsterdam Modeling Suite program, Amsterdam Density Functional (ADF).[2] Geometry optimizations of neutral, anionic, and cationic aluminum clusters ranging from 3 - 8 atoms were performed at the generalized graduent approximated Perdew, Becke, and Erzerhof (GGA-PBE) exchange-correlation functional and triple zeta with polarization functions (TZP) Slater type basis set level of theory with no frozen core.

	Top View	Side View
LUMO	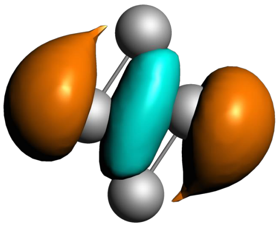	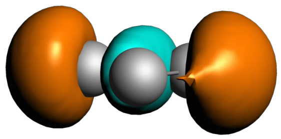
HOMO	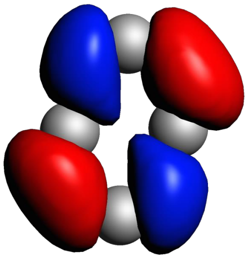	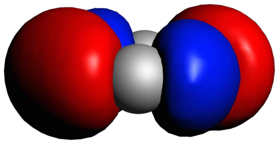

Figure S1: Top View and Side View of Highest Occupied Molecular Orbital and Lowest Unoccupied Molecular Orbital of Al_4

References

1. Rao, B.; Jena, P. Evloution of the electronic structure and properties of neural and charged aluminum clusters: A comprehensive analysis. *The Journal of chemical physics* **1999**, *111,* 1890-1904.

2. te Velde, G.; Bickelhaupt, F. M.; Baerends, E. J.; Fonseca Guerra, C.; van Gisbergen, S. J. A.; Snijders, J. G.; Ziegler, T. Chemistry with ADF. J. Comput. Chem. **2001**, *22*, 931 - 967.
